# Estimating the societal cost of type 2 diabetes in Malmö, Sweden: a register-based cost analysis

**DOI:** 10.3389/fcdhc.2025.1611426

**Published:** 2025-12-03

**Authors:** Magdalena Annersten Gershater, Alexander Dozet, Åsa Ericsson, Slobodan Zdravkovic

**Affiliations:** 1 Department of Care Science, Faculty of Health and Society, Malmö University, Malmö, Sweden; 2 Corporate Office, Region Skåne, Malmö, Sweden; 3 National Market Access & Public Affairs, Novo Nordisk, Malmö, Sweden

**Keywords:** cardiovascular disease, cost analysis, diabetes complications, diabetes mellitus, diabetic retinopathy, diabetic kidney disease, healthcare economics and organisation, health economic modelling

## Abstract

**Background:**

With the prevalence of Type 2 Diabetes (T2D) projected to increase, understanding its potential consequences on healthcare systems is crucial for adequately preparing society to address this growing challenge. In 2019, Malmö, Sweden’s third-largest city, joined the Cities for Better Health global initiative to tackle the multifaceted challenges associated with T2D, including its significant economic burden on the healthcare system and the broader community. Understanding the economic burden of T2D on the healthcare system will facilitate optimisation of the initiatives undertaken by the programme. Therefore, this study aimed to estimate the costs associated with primary care, hospital care, and work absenteeism due to diabetes-related complications among people with T2D residing in Malmö.

**Methods:**

In order to estimate the cost for the City of Malmö, we expanded a model (Andersson et al., 2020) developed to estimate the cost of T2D on a national level, using retrospective data from 1997–2016. The costs were estimated by using NordDRG weights and national reference prices. Primary care costs for Region Skåne were added to the model. Data on healthcare utilisation, work absence, and socioeconomic factors were collected from Swedish national and regional registers. The method was expanded to include Malmö-specific adjustments for demographics, employment, and education, as well as regional primary care costs.

**Results:**

The prevalence of T2D in Malmö was 5.4%, and diabetes complications were: diabetic retinopathy (49.9%), diabetic kidney disease (19.1%), angina pectoris (13.7%), ischaemic heart disease (10.9%), and myocardial infarction (10.5%). Total excess costs for T2D in primary care were €12.7 million. The lowest primary care excess costs were in the age group 16–34 and the highest in the age group 65–74. Estimated overall hospital-based costs for T2D were €38.8 million, and costs related to macrovascular and microvascular complications were €18.1 million and €16.4 million, respectively. Estimated total cost due to absence from work related to T2D complications was €15.4 million. The complication costs were higher for men, except for neuropathy.

**Conclusions:**

These findings may support city-level healthcare planning and preventive interventions, as Malmö is facing substantial costs both in monetary terms and in reduced quality of life. Younger persons increasingly develop diabetes complications, which needs to be considered when allocating resources for primary prevention, treatment of complications, and municipality costs within a near future.

## Highlights

- The prevalence of T2D in 2016 for persons residing in Malmö was 5.4%.
- The most common complications were diabetic retinopathy (49.9%), cardiovascular disease (IHD, angina pectoris, acute myocardial infarction, stroke, heart failure, and atrial fibrillation) (35.1%), and diabetic kidney disease (19.1%).
- The total excess costs for T2D in primary care were €12.7 million; estimated overall hospital-based costs for T2D were €38.8 million; and estimated total costs due to absence from work related to T2D complications was €15.4 million.
- This represents an ongoing public health challenge. As diabetes complications increasingly affect younger individuals, it is essential to allocate resources towards primary prevention, effective management of diabetes and complications, and the anticipated rise in municipal healthcare expenditures.

## Background

The prevalence of type 2 diabetes (T2D) is increasing worldwide and is expected to increase even further over the coming decades (1). This trend affects healthcare organisations in terms of rising direct healthcare costs for the treatment of T2D and its complications. The costs include both hospital-based care and primary care, as well as productivity loss due to absence from work and, consequently, decreased tax revenue (2). Steen Carlsson et al. (3) estimated the total costs of T2D in Sweden to be €1.79 billion in 2013 and predicted the cost to be €2.35 billion in 2030.^1^ Only 23% of these costs were related to diabetes prevention, whereas diabetes complications accounted for 70%. A more recent register-based study from Sweden revealed that the costs of hospital-based care and absence from work related to diabetes complications amounted to €1,153 million (€2,943 per person with diabetes) in 2016, of which hospital-related costs accounted for €269 million and indirect costs for €884 million (4). In addition, it has been estimated that implementing a treatment regimen to lower HbA1c, in accordance with the Swedish national treatment guidelines, would be cost neutral. Such an approach could save approximately 4,000 life years and prevent an estimated 800 myocardial infarctions, 500 strokes, 450 cases of end-stage renal disease requiring dialysis, and 200 cases of blindness by 2030 (3). It is critical to take into account the challenges posed by the progressive nature of T2D, its complications and its increasing prevalence when planning and structuring an adequate healthcare system for the coming decades. As the number of people living in urban settings is growing, Novo Nordisk, University College London, and the Steno Diabetes Centre Copenhagen launched a cross-organisational collaboration initiative in 2014 – Cities Changing Diabetes (5), renamed Cities for Better Health in 2024. Currently, there are 51 cities on five continents included in the programme. In 2019, the programme was extended to include the City of Malmö, Region Skåne, as the first Swedish city (https://www.citiesforbetterhealth.com/network/our-cities/malmo.html). The aim is to break the increasing prevalence of T2D and support the healthcare system, as well as promoting health-related factors on a local level, as Malmö is a socioeconomically diverse and segregated city with a young population. In a first survey of T2D in Malmö, the prevalence had doubled between 2011 and 2018, and the prevalence was unevenly distributed in the city, with more cases in socioeconomically deprived areas (5, 6). The prevalence was also higher in the older population. As an immediate consequence of the survey, the City of Malmö appointed a diabetes nurse to coordinate education for healthcare staff in the home care organisation. The Cities for Better Health initiative is particularly relevant for Malmö, given its diverse demographic and socioeconomic challenges, including a rising prevalence of T2D, and its commitment to addressing the long-term health implications for vulnerable populations. The city’s high rates of migration increase these challenges, leading Malmö to consider the future consequences for home care services and the growing number of older people facing health issues. By joining Cities for Better Health, Malmö aimed to learn from other cities with similar challenges, as this global initiative fosters collaboration and the sharing of best practices to more effectively combat the impact of diabetes. Three action groups have been created in Malmö to identify activities for meeting citizens’ needs in terms of prevention and care: 1) prevention for children and young people, 2) prevention for adults, and 3) equal diabetes care for all patients. The present study supports these action groups aimed at developing in developing relevant interventions.

The local government structure in Sweden is organised into 21 regions and 290 municipalities. The City of Malmö, with a population of 348,000, is the largest municipality in Region Skåne, which is Sweden’s southernmost region, with a total population of 1.39 million (7). The main responsibility for the region is to provide hospital and primary healthcare, whereas the municipalities have a wider range of responsibilities, such as home-based nursing and older adult care (8). However, the delineation of responsibility for healthcare between the regional and municipality levels is not always straightforward. There are overlapping responsibilities, which may result in inefficiencies from a societal perspective. The two levels of local government are responsible for their own budgets and are funded independently – a division that could lead to a situation where the costs of certain activities, for example, prevention (primary care) and treatments of diabetes complications (hospital), are borne solely by the region, while the benefits are accrue to municipalities that do not contribute to the funding of those activities. This could jeopardise the viability of the activities, even though they may be desirable from a societal perspective. The municipalities do, however, carry the burden of caring for patients with complications until the end of life.

At the time of the present study, there were 38 primary care centres and one hospital (www.1177.se) in Malmö. The primary care centres are reimbursed based on the number of listed patients, while the hospital is reimbursed by means of a national budget. Physicians, registered nurses, and other healthcare staff are paid a monthly salary and have no direct financial interests in the treatment offered to patients. The current growth in population is mainly attributed to migration to Malmö from other parts of the country as well as from abroad, in combination with high birth rates. With almost 50% of the population under the age of 35, the city has a young population. A third of the population was born outside of Sweden (9).

Statistic Metropolitan Malmö is expected to follow the worldwide trend, it is important to raise awareness of the potential economic impact of such a development on healthcare. Furthermore, since Sweden’s healthcare system is decentralised to the regional level, any action to manage T2D must be initiated at that level. However, resources for conducting localised estimates of T2D’s impact on healthcare utilisation and costs are limited. In the absence of locally tailored estimates—and given the similarities in healthcare systems across regions in Sweden—it is feasible to use studies from other regions or national-level studies, with adjustments made for the local population.

Therefore, the aim of the current study was, using an already developed national model (4), to estimate primary care and hospital-based costs (outpatient and inpatient care) as well as costs relating to work absence due to complications in people with T2D residing in Malmö.

## Materials and methods

To estimate the cost of T2D in Malmö, we adopted a model presented in Andersson et al. (4), where the cost associated with diabetes complications was based on a retrospective data study drawing on 20 years of individual data from 1997 to 2016.

### Population

The entire Malmö population with T2D, from 16 years of age, was selected and categorised according to sex and age, using data from the Swedish National Diabetes Register (NDR). The costs for hospital-based care were calculated by applying the cost from the national level (4) estimated with adjustments to the observed demographic structure of the Malmö population, educational level, and employment rate. The costs for primary care in Malmö were added. Classification of diabetes was done using the ICD10-codes E11 and E14. The prevalence of T2D was calculated as the ratio between the number of persons diagnosed with T2D and living in Malmö in 2016 and the total number of people living in the city that year.

### Data sources

The data were collected from three Swedish national authorities: the National Board of Health and Welfare, the Swedish Social Insurance Agency, and Statistics Sweden. Socioeconomic data were obtained from Statistics Sweden’s Longitudinal integrated database for health insurance and labour market studies (LISA). Data on work absence were obtained from the Swedish Social Insurance Agency’s database, Micro-Data for the Analysis of Social Insurance (MiDAS), that covers sickness and rehabilitation benefits.

To define complications, ICD-10 and procedure codes were used, as presented in the electronic supplementary material summarised in **Table 1** in Andersson et al. (4). People with T2D and diabetes-related complications, were identified through medical visits recorded in the National Patient Register (7), which includes outpatient visits and inpatient hospitalisation, as well as through prescriptions for glucose-lowering medications found in the National Prescribed Drugs Register (10). The latter covers all drugs within the pharmaceutical benefits scheme that are prescribed and dispensed at pharmacies.

**Table 1:** Frequencies and per cent of T2D complications in Malmö and Sweden.

Complications	Malmö (n=14,324)	Sweden (n=476,728)
Eye disease and diabetic retinopathy	7,141 (49.85)	24,5243 (51.44)
Diabetic kidney disease	2,738 (19.12)	92,584 (19.42)
Angina pectoris	1,958 (13.67)	69,353 (14.55)
Ischaemic heart disease	1,567 (10.94)	55,409 (11.62)
Acute myocardial infarction	1,500 (10.47)	53,061 (11.13)
Atrial fibrillation	1,308 (9.13)	46,940 (9.85)
Stroke	1,145 (8.00)	40,673 (8.53)
Heart failure	1,035 (7.23)	36,404 (7.64)
Neuropathy	886 (6.18)	29,366 (6.16)
Peripheral vascular disease	824 (5.75)	29,208 (6.14)
Diabetic foot and ulcer	791 (5.52)	26,813 (5.62)
Osteoarthritis	728 (5.08)	26,364 (5.51)
ESRD with dialysis or kidney transplantation	203 (1.42)	6,478 (1.36)
Amputation	160 (1.12)	5,695 (1.19)
Hypoglycaemia	124 (0.87)	4,185 (0.88)
Hyperglycaemia	110 (0.76)	3,577 (0.75)
Ketoacidosis	19 (0.13)	586 (0.12)
Coma	13 (0.09)	432 (0.09)
Vision loss or blindness	60 (0.42)	1,973 (0.41)
Other sudden death, cause unknown	0	0

### Cost estimation

The study focused on healthcare use and work absence (both short- and long-term absence) to estimate the cost of diabetes complications. For each person with the diagnosis T2D, five control persons from the general population were matched based on birth year, gender, and region of residence. Diagnosis-related group (DRG) codes and national prices for 2016 were used to calculate the healthcare cost. The cost of work absence was estimated by multiplying absence days with age- and gender-specific income levels. The cost of mortality in working ages was calculated using the human capital approach (4). As to the cost of T2D in Malmö, it was estimated by extrapolating data. Malmö-specific weights were calculated to adjust for differences from the national Swedish population, using factors such as sex, age, employment status, and education level, as outlined by Steen Carlsson et al. (3). The model used assumed a consistent complication risk across these factors in Malmö. This study estimated costs for primary and hospital care, as well as work absences due to T2D complications, for the year 2016. However, costs for transportation, time, self-care equipment, home nursing, and assisted living facilities were not included in the present study. Andersson et al. used DRG codes with contact-specific weights from NordDRG (4). Demographic and socioeconomic variables were cross-linked to the persons from Statistics Sweden’s databases (www.scb.se).

### Primary care

In the current paper, the cost estimation was expanded to also include the cost of primary care, which was not included in the national estimation due to the lack of a national register of primary care. Healthcare cost data for primary care in Malmö were obtained from the regional register Patient Administrative System in Region Skåne (PASiS). By using the healthcare cost data, the costs were estimated for each person with T2D and categorised by age group. All costs were converted to euros (EUR) using the 2023 exchange rate (11, 478 Swedish kronor per euro) and adjusted for inflation based on Sweden’s 2023 Consumer Price Index (CPI).

## Results

### Prevalence of T2D

**Figure 1:**
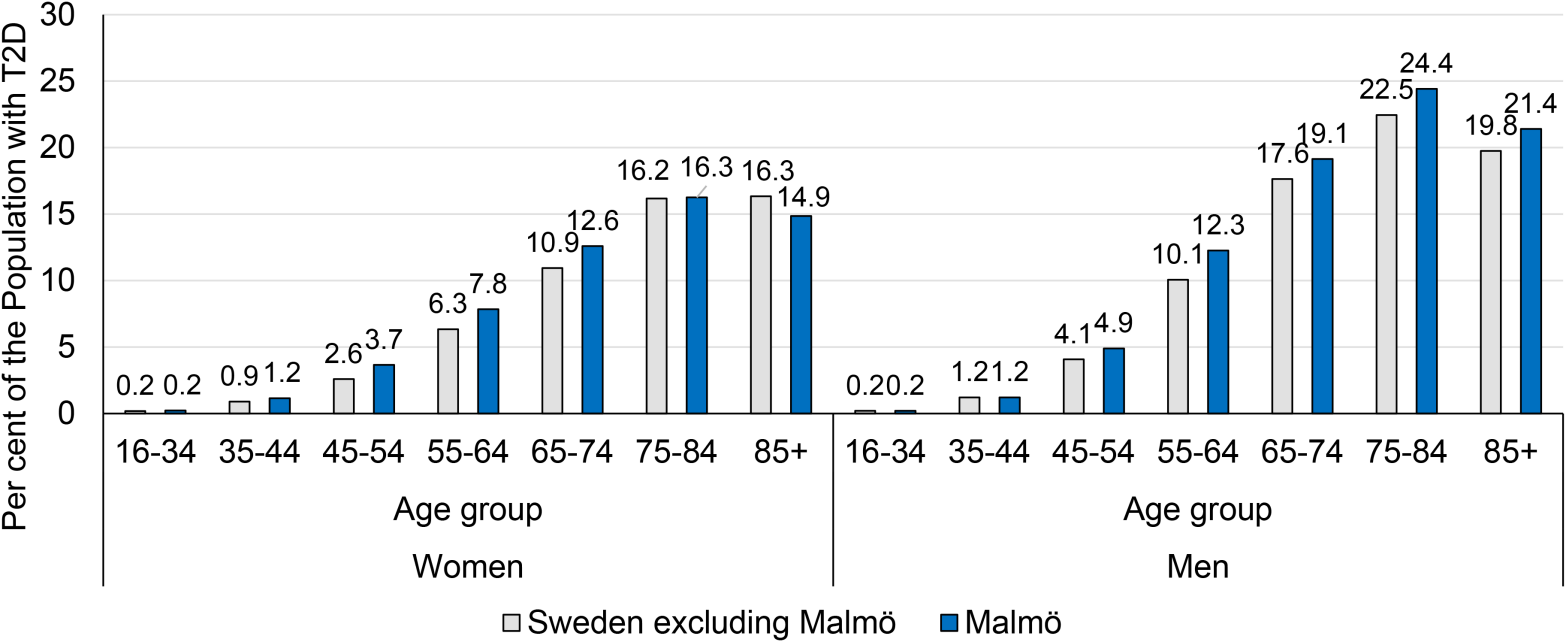
T2D prevalence by gender and age group for Malmö and for Sweden with Malmö excluded.

The total number of individuals in the population of Malmö being 16 years of age or older was N = 269,581. The observed prevalence of T2D in 2016 for persons aged 16 years or over residing in Malmö was 5.4%. The prevalence was higher among men than among women (4.7% among women and 6.1% among men). The lowest T2D prevalence (0.2%) was observed in the age group 16–34, among both men and women (**Figure 1**).

### Complication burden

The ratio of number of complications per person was 1.54, as 14,324 people with T2D had 22,310 complications. The most frequent type of complication in both men and women was microvascular complications, which occurred more often in men (n=6,328) than in women (n=5,684).

**Figure 2:**
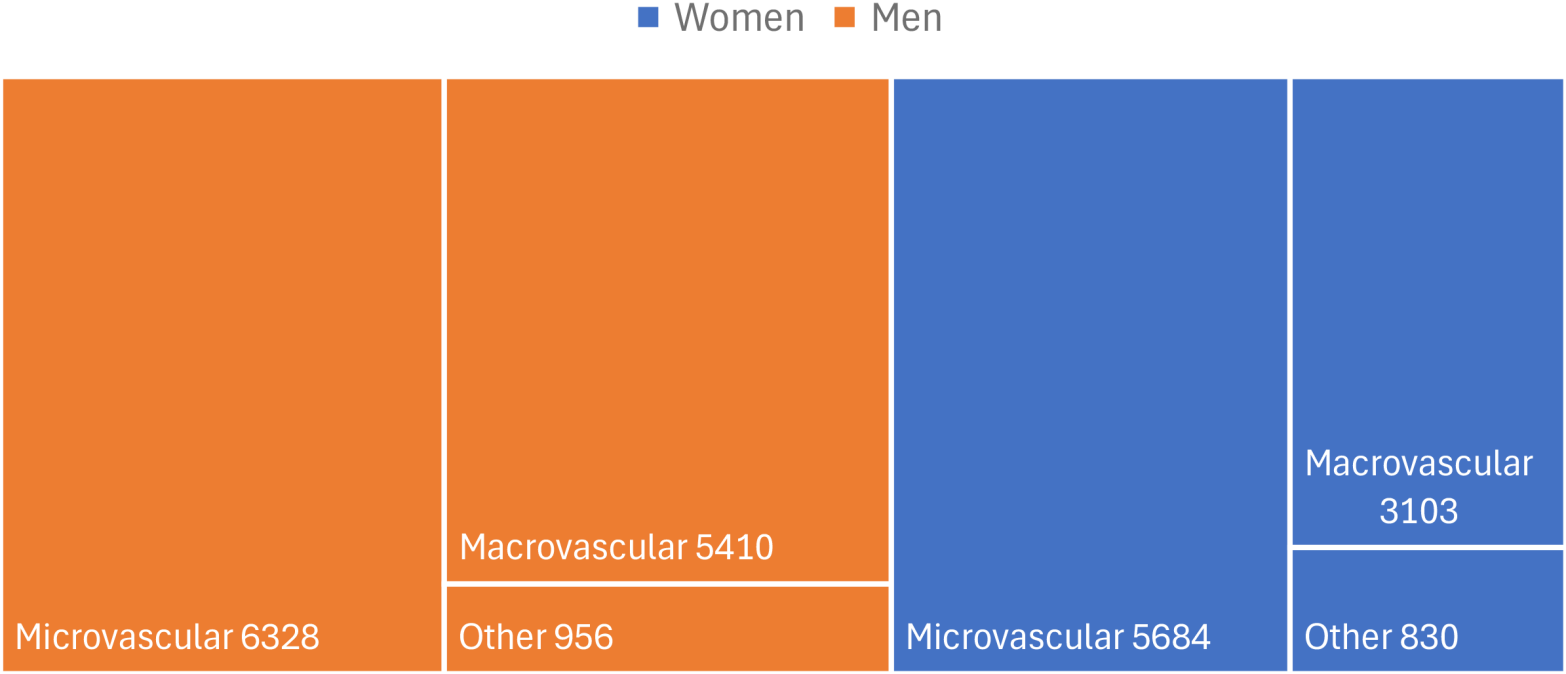
Frequencies of T2D complications (n=22,311) grouped as microvascular, macrovascular, and other, in Malmö by gender. *A tree map showing the proportion of each type of complication in relation to the total number of complications.

The five most common T2D specific complications in Malmö were diabetic retinopathy (7,141), diabetic kidney disease (2,738), angina pectoris (1,958), ischaemic heart disease (1,567), and myocardial infarction (1,498) (**Table 1**). Frequencies of all micro- and macrovascular complications are presented in **Figure 2**.

### Costs

The total cost of all hospital-based care in Malmö was €146 per capita (compared to all hospital-based care in Sweden – excluding Malmö – where the total cost was €167 per capita).

#### Primary care

The total excess costs for T2D in primary care were €12.7 million (€6.1 million for women and €6.6 million for men). The lowest primary care excess costs were observed in the age group 16–34, with the highest costs in the age group 65–74 (**Table 2**).

**Table 2:** Total excess primary care costs (€, million, adjusted for CPI 2023) for type 2 diabetes, by age group and gender.

Age category	Men Cost (n)	Women Cost (n)	Total Cost (n)
16–34	0.041 (94)	0.063 (109)	0.104 (203)
35–44	0.225 (307)	0.224 (261)	0.45 (568)
45–54	0.739 (966)	0.747 (693)	1.487 (1,659)
55–64	1.612 (1,992)	1.303 (1,284)	2.915 (3,276)
65–74	2.068 (2,460)	1.795 (1,791)	3.863 (4,251)
75–84	1.423 (1,600)	1.402 (1,451)	2.824 (3,051)
85+	0.456 (527)	0.588 (789)	1.044 (1,316)

#### Hospital-based care

The estimated overall hospital-based costs for T2D were €38.8 million (€16.0 million for women and €22.8 million for men). The overall healthcare costs related to macrovascular and microvascular complications were €18.1 million and €16.4 million, respectively (**Table 3**). Macrovascular complication costs were €1.40 million among women and €3.54 million among men, and microvascular complication costs were €3.74 million among women and €4.71 million among men. The six complications with the highest costs were diabetic retinopathy, diabetic kidney disease, stroke, neuropathy, angina pectoris, and diabetic foot.

**Table 3:** Hospital-based costs^1^ per T2D complication (€, million, adjusted for CPI 2023).

Cost for macrovascular complications	Cost for microvascular complications	Cost for other complications
Heart failure 6.104	Diabetic kidney disease 4.03	Arthrosis 3.222
Stroke 3.371	Diabetic Retinopathy 3.589	Hypoglycaemia 0.525
Myocardial infarction 3.291	Peripheral vascular disease 3.269	Hyperglycaemia 0.197
Angina pectoris 2.901	End-stage renal disease 3.07	Keto acidosis 0.159
Ischaemic heart disease 1.351	Amputation 1.310	Coma 0.074
Atrial fibrillation 1.1	Diabetes-related ulcers 1.082	Sudden death^2^ 0
Total 18.12	Total 16.35	Total 4.177

^1^ The costs were calculated based on DRG codes and national prices for 2016. Complications were classified by the ICD codes, as presented in Andersson et al. 2020.

^2^ Zero cost due to no attributable hospital cost.

Colour explanation: red > 4 million, pink 2–3.9 million, yellow 1–1.9 million, and beige < 1 million.

The costs per person increase by age except for the age group 35–44. The highest cost per person with T2D was observed in the age group 85+ among both women and men (€10.218 for women and €10.227 for men). The lowest cost per person was observed in the age group 35–44 for women (€980) and in the age group 35–44 for men (€1,273).

#### Productivity loss costs

The estimated total costs due to absence from work related to T2D complications were €15.4 million (€5.89 million among women and €9.46 million among men). The costs were higher for men across all complications except for neuropathy, where the cost for women (€0.969 million) exceeded that for men (€0.679 million).

#### Health care costs

The simulated excess healthcare costs due to T2D complications increased with increasing age up to 84 years for women and 74 for men. The highest excess costs both for primary care and for hospital-based care were observed in the age group 75–84 for women and in the age group 65–74 years for men. The excess cost for primary care was €1.79 million for women (age group 65–74) and €2.07 million for men (age group 65–74). For hospital-based care, the highest estimated excess care cost was €5.0 million in the age group 75–84 for women and €7.18 million in the age group 65–74 for men. The lowest excess hospital-based and primary care costs were observed for persons between 16 and 34 years of age. The highest excess costs due to productivity loss were observed in the age group 55–64 in both women and men (**Table 4**).

**Table 4:** Excess costs due to T2D complications in Malmö (€, million, 2023), by age group and gender.

Gender	Age group	Primary care	Hospital care	Productivity loss	Total cost
Women	16–34	0.063	0.071	0.145	0.279
	35–44	0.224	0.192	0.446	0.862
	45–54	0.747	0.686	1.585	3.018
	55–64	1.303	1.707	3.717	6.727
	65–74	1.795	3.775	0	5.570
	75–84	1.402	4.990	0	6.392
	85+	0.588	4.548	0	5.136
Men	16–34	0.041	0.083	0.141	0.265
	35–44	0.225	0.293	0.539	1.057
	45–54	0.739	1.187	2.202	4.128
	55–64	1.612	3.594	6.578	11.784
	65–74	2.068	7.183	0	9.251
	75–84	1.423	6.975	0	8.398
	85+	0.456	3.478	0	3.934

**Figure 3:**
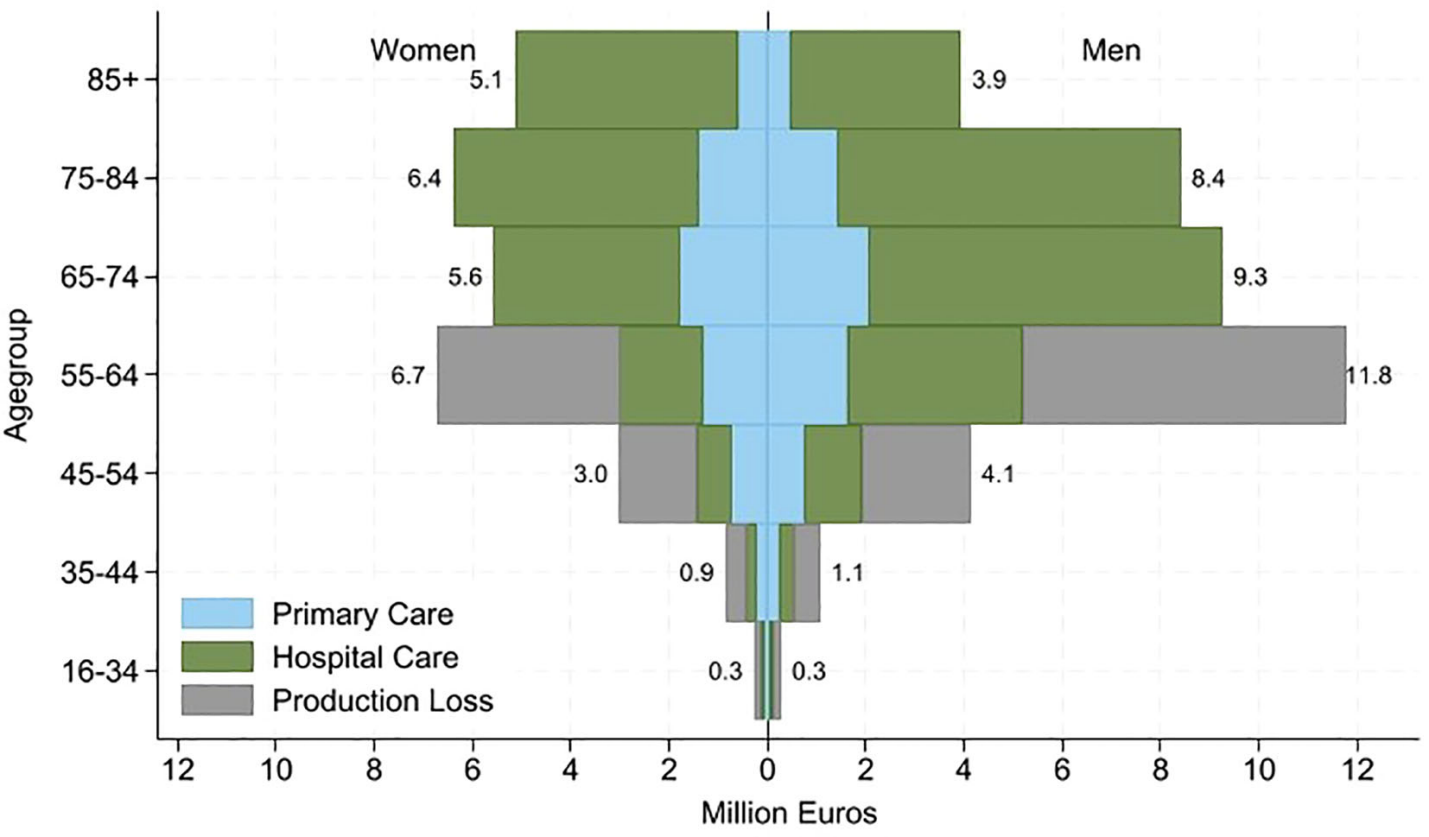
Decomposition of excess costs (€, 2023) for T2D patients for primary care, hospital care, and productivity loss, by age and gender, adjusted for CPI 2023 in millions.

A graphical illustration of excess costs is presented in **Figure 3**.

## Discussion

The rising prevalence of T2D in Malmö will likely lead to an increased number of persons living with various T2D-related complications, which may result in higher costs for both healthcare organisations and patients. As the population projection for Malmö indicates that the number of children, adolescents, and older persons will increase, this will lead to a higher prevalence of T2D, since young people with T2D (11) live longer with the disease, which in turn may increase the risk of diabetes-related complications.

In the present study, the prevalence of diabetes-related complications is high, with the two most common complications being diabetic retinopathy and diabetic kidney disease. This indicates that diabetes management might need improvements (12, 13). Targeted prevention programmes and early detection and treatments of T2D, including cardiovascular risk factors, are therefore important in order to reduce the risk of future micro- and macrovascular complications and early death (12, 13). This requires optimal treatment at an early stage, including medications lowering HbA1c, blood pressure, and lipids, in addition to physical activity and a healthy diet (12). Compared to the past two decades, medical treatments as well as healthcare systems have evolved to include a considerably wider range of treatment options, such as multidisciplinary clinics, SGLT2-inhibitors, GLP1-RA, and an increased use of CGM, as presented by the American Diabetes Association in 2024 (14). T2D is a progressive disease characterised by increased levels of glycated haemoglobin (HbA1c). Prolonged periods of poor glycaemic control are associated with an increased risk of macro- and microvascular complications, resulting in substantial treatment costs (13, 15–17). Medication is largely reimbursed by the regions in Sweden, but access to diabetes education in Malmö is difficult since no healthcare provider offers group education for these patients, and there is no structured diabetes self-care education programme (12, 14). The lack of structured educational programmes might have a negative impact on patients’ health-related empowerment and on other prevention efforts to minimise health deterioration and complication progress. This is an area that needs to be developed by the healthcare organisations (both in primary care and in specialist clinics), as it is critical for the patients’ ability to perform adequate self-care and thereby achieve their treatment targets. There is a regional certification system for healthcare centres regarding the quality of diabetes care (18); however, only eight out of 38 healthcare centres have this certification (Personal communication with diabetes coordinator 5 Sept. 2022). In addition, there is no public information for the patients about where to find a diabetes-certified healthcare centre in Malmö.

In the present study, the five complications with the highest costs, besides diabetic retinopathy, were neuropathy, stroke, diabetic kidney disease, foot ulcers, and angina pectoris. The costs of T2D and its complications are substantial in monetary terms, and may result in a sustained reduction in quality of life (19). Thus, in Malmö – as in other cities – diabetes leads to considerable costs for both primary care and hospital care. There are also costs for municipal home care, costs that were excluded in the present study, however. The shared responsibility for diabetes care between the Region (medical care), the Municipality (assisted self-care), and the State (reimbursement for sick leave for people working) entails some difficulties. Hence, the division of the Swedish healthcare system may result in inefficiency, since regions and municipalities operate independently with separate budgets and documentation systems. In terms of absence from work, shared responsibilities have consequences for the whole society. Direct costs for treating diabetes and its complications are higher for male patients, who use more hospital resources and primary care. Men also have more absence from work due to diabetes. The reasons for this are probably multifactorial but some explanations have been proposed in previous studies, including hormonal factors, suggesting that oestrogen may have a possible effect on vascular complications (20). In addition, men and women differ in attitudes towards self-care, with women being more likely to care for their health (21, 22).

The present study revealed that the TD2 population in Malmö is younger than in Sweden as a whole. Regardless of gender, the T2D prevalence in Malmö was 17% (age group 16–54), compared to 13% nationally for the same age group (11). Many experience complications before the age of retirement. Hellgren et al. stated that the economic burden of diabetes could be reduced by early and effective treatment to lower blood glucose (19). However, with the same level of sustained high Hba1c-levels, increased resources need to be allocated in the near future for treatment of diabetes complications in relatively young persons with T2D.

Some of the health-related costs are due to the need of home care efforts for this patient group. For example, 17% of patients with a first foot ulcer already have home nursing or live in assisted living at the time of the ulceration (23). Moreover, in Swedish municipal home care, 10% of those receiving care are treated with blood glucose-lowering agents and 20% of those with diabetes have ongoing foot ulcers (24). In this study, it would therefore have been valuable to include additional costs for persons with multiple diabetes-related complications – that is, costs for transportation, time, self-care equipment, home nursing, and assisted living facilities. However, including these costs in the present study was not feasible.

Since the Swedish healthcare system follows a shared structure across regions, costs can be estimated for different regions, including Malmö, by utilising national-level data that have been adjusted to account for the population characteristics of Malmö. One value of this is that it can form the basis for policy decisions for the future prioritisation of prevention activities. The highest excess cost due to T2D was observed in the age group 65–74 years combining primary care and hospital-based care. This implies that newly retired persons with diabetes live with substantial disabilities that could have been prevented by a later onset of T2D and early optimised treatments, delaying the development of diabetes complications. Furthermore, the highest hospital-based cost per person was observed among those above the age of 85 years, which might be due to the higher level of severity of established comorbidities in that age group. In particular, the high prevalence of diabetic retinopathy and diabetic nephropathy indicates that preventive treatments can be improved within primary care in Malmö.

These findings underscore the need to prioritise preventive and therapeutic interventions for younger people at risk of T2D, in order to reduce disability and long-term healthcare costs as they age. Furthermore, without screening programmes and early diagnosis, the increase in younger people with T2D could lead to greater productivity losses, which may in turn affect the Swedish social insurance system. The findings could help inform health resource allocation in Malmö and in Sweden as a whole, for early systematic screening of specified high-risk target groups, diabetes awareness campaigns, and improved diabetes competency in primary care with diabetes specialist nurses and general practitioners. Such resource allocation is expected to reduce the incidence of T2D as well as improving health equity. Through initiatives such as Cities for Better Health, different stakeholders can collaborate in developing Malmö-specific activities to delay the onset of T2D and its consequences for the population. In Malmö, the initiative currently involves interventions aimed at preventing diabetes both for children and young people and for adults, as well as efforts to ensure equitable diabetes care for all Malmö residents. The strategies employed in Malmö could provide valuable insights for developing comparable public health initiatives in other urban areas experiencing an increase in T2D prevalence.

### Limitations

The NDR is a Swedish register including the mapping and development of diabetes care in Sweden. However, one limitation in the present study is that no data exist that are specifically linked to costs for diabetes in the City of Malmö Health and Social Care Department, which means that an important part of the cost structure could not be obtained, namely, that of home-based healthcare. Another limitation is that, for the estimation of costs, the national cost structure was applied to Malmö. If unemployment rates, salaries, or healthcare unit costs differ between Malmö and Sweden as a whole, this might skew the results. Furthermore, when calculating costs for diagnosis-related groups, there are elements of standard calculation. Thus, the costs are administratively set prices based on national-level data to estimate the resource use costs, and these unit costs may differ at the local level. Moreover, a limitation of register studies is the absence of indirect costs, such as informal care provided by family members.

The 21 regions in Sweden share many similarities in the cost structure and organisation of the healthcare system. We therefore estimated the cost impact of T2D in the City of Malmö by applying a national cost estimation and adjusting for demographic, educational, and employment differences between the population of Malmö and that of the whole of Sweden. Furthermore, we improved the cost estimation by including primary care costs, which were not available in the national estimation. This study suggests that the use of nationally collected data, when adjusted for population differences, may be sufficient to estimate the scale of the resource burden and provide a basis for creating health policy regarding T2D, for smaller geographical areas participating in Cities for Better Health. However, local nuances, such as access to care and the integration of social services, could still restrict the generalisability of these findings.

## Conclusions

The high costs and complications related to T2D in Malmö highlight an urgent need for proactive measures to mitigate the financial burdens on healthcare and protect the quality of life for those at risk of the disease. Moreover, the proportion of young people with T2D is larger in Malmö than in the rest of the country, and they face a high risk of developing diabetes-related complications over time. The findings from this study may therefore inform future decisions about resource allocation for primary prevention, treatment of complications, and municipality costs in the near future. The Cities for Better Health initiative can convey the available facts to local stakeholders in order to support the development of targeted cross-disciplinary and cross-sector public/private partnership interventions. The results of this study may help guide the Cities for Better Health project towards areas where the needs – and the potential value of social initiatives – are greatest. Given the high prevalence of T2D among young people in Malmö, it is essential to continue conducting research and monitoring efforts. By doing so, society may be able to effectively adapt strategies to address the evolving burden of this condition and work towards improved health outcomes for future generations.

## Data Availability

The raw data supporting the conclusions of this article will be made available by the authors, without undue reservation.

## Footnotes

1 All costs were converted from Swedish kronor (SEK) in 2016 to euros (EUR), using the exchange rate for the year 2023 and adjusted for inflation according to the Swedish Consumer Price Index for 2023.
